# Editorial: Health-related quality of life among Hodgkin lymphoma survivors

**DOI:** 10.3389/fonc.2025.1625160

**Published:** 2025-06-06

**Authors:** Esther Natalie Oliva, Alberto Fabbri, Àrpàd Illès

**Affiliations:** ^1^ London North West University Healthcare NHS Trust, London, United Kingdom; ^2^ Unit of Hematology, Azienda Ospedaliera Universitaria Senese and University of Siena, Siena, Italy; ^3^ Division of Hematology, Department of Internal Medicine, University of Debrecen, Debrecen, Hungary

**Keywords:** Hodgkin lymphoma, HRQL = health-related quality of life, survivorship, hematology malignancies, cognitive impairment

The Research Topic *Health-related quality of life among Hodgkin lymphoma survivors* in *Frontiers in Oncology* delves into the unique challenges faced by individuals who have overcome Hodgkin lymphoma (HL), particularly in sustaining a high quality of life (QoL) post-treatment. While modern therapeutic approaches have significantly improved survival rates, long-term survivors often experience lingering health complications, including fatigue, cognitive dysfunction, and social difficulties.

The article “*Longitudinal study of cognitive and mental functions among adult Hodgkin-lymphoma survivors*” provides valuable insights into the cognitive and mental health challenges faced by Hodgkin lymphoma survivors over time (Virga et al.). One key strength of the study is its longitudinal approach, which allows for a more comprehensive understanding of how cognitive and mental functions evolve post-treatment. Previous studies often rely on cross-sectional data, which may overlook patterns in long-term survivorship care. Additionally, the authors’ analysis, based on data from a Hungarian treatment center, presents valuable regional specificity. However, a notable limitation is its limited generalizability, as findings from a single treatment center may not fully capture variations across diverse healthcare systems and demographic groups. Future research could expand on this by incorporating multi-center studies or comparing results across different populations. This study further reinforces the necessity of psychological support for HL survivors, as cognitive dysfunction and mental health concerns can significantly affect overall well-being. Integrating mental health services into routine follow-ups could prove beneficial.

The study “*Risk factors analysis and the establishment of nomogram prediction model for PICC-related venous thrombosis in patients with lymphoma*” explores risk factors for peripherally inserted central catheter (PICC)-related venous thrombosis in lymphoma patients while proposing a predictive model for enhanced clinical decision-making (Wang et al.). A standout feature of this research is its double-center, cohort-based case-control design, which enhances the reliability of the findings by incorporating data from multiple sources. The “nomogram prediction model” introduced in this study is particularly valuable, as it provides clinicians with a practical tool to assess thrombosis risk and adjust treatments accordingly. Nevertheless, generalizability remains a concern, as further validation across diverse clinical environments could enhance its applicability. While the study effectively identifies thrombosis risk factors, future research could explore preventive strategies aimed at reducing complications in lymphoma patients undergoing PICC placement. Overall, this article emphasizes the importance of personalized risk assessment and multidisciplinary collaboration among oncologists, hematologists, and vascular specialists to optimize patient care.

The systematic review “*Patient-reported outcomes in Hodgkin lymphoma trials: a systematic review*” offers a broad analysis of how “patient-reported outcomes (PROs)” are integrated into clinical trials for HL treatment (Olivia et al.). One of the strengths of this study is its systematic approach, which synthesizes data from multiple trials to provide a broad perspective on PROs in Hodgkin lymphoma research, underscoring their importance in evaluating the full impact of treatment beyond traditional clinical metrics. While the review provides meaningful insights, the lack of standardization in PRO measures across trials remains a challenge. Establishing consistent methodologies could strengthen data comparability and ensure that patient perspectives play a more significant role in clinical decision-making. Future studies could also focus on longitudinal PRO assessments, tracking patients’ PRO changes over time. Ultimately, this review advocates for holistic survivorship care, ensuring that clinical trials emphasize both treatment efficacy and long-term well-being.

The review “*Bone damage and health-related quality of life in Hodgkin lymphoma survivors: closing the gaps*” examines the overlooked issue of bone health in HL survivorship, emphasizing its impact on QoL (Mancuso et al.). A key strength of this study is its comprehensive assessment of osteopenia and osteoporosis in HL survivors, shedding light on treatment-induced bone deterioration—an area that often lacks sufficient attention in post-treatment care. However, a key concern is the absence of standardized screening protocols for bone health among lymphoma survivors. While this study effectively discusses the correlation between cancer therapy and bone damage, future research could propose targeted interventions, such as exercise regimens and nutritional strategies, to help mitigate these effects. In essence, this article highlights the importance of multidisciplinary survivorship care, ensuring that health complications beyond cancer remission are effectively addressed.

In summary, emerging research underscores the necessity of longitudinal studies to track how HRQoL evolves among HL survivors. Bridging this gap could enable more targeted interventions to support survivors’ “physical, mental, and social health”. Furthermore, comprehensive survivorship care must extend beyond routine medical evaluations. HL survivors require “psychological support, lifestyle guidance, and social reintegration strategies” to foster overall well-being. The incorporation and reporting of PROs in clinical trials is also pivotal in ensuring that treatment decisions prioritize “long-term QoL” alongside remission success.

Ultimately, this Research Topic serves as a “call to action” for healthcare providers, researchers, and policymakers to refocus efforts on survivorship care beyond treatment success. By deepening our understanding of HRQoL in HL survivors, the medical community can work toward integrated care models that support patients long after remission.

There is an emerging importance of longitudinal studies in assessing the evolving health-related quality of life (HRQoL) of HL survivors. Most existing research relies on retrospective or cross-sectional studies, leaving gaps in understanding how survivors’ well-being changes over time. Addressing these gaps could lead to more targeted interventions that improve physical, mental, and social health outcomes.

Additionally, there is a need for holistic survivorship care. Beyond medical follow-ups, HL survivors require psychological support, lifestyle guidance, and social reintegration strategies to enhance their overall well-being. The integration of patient-reported outcomes (PROs) in clinical trials is also crucial to ensure that treatment decisions prioritize long-term quality of life (Virga et al.).

